# Estimation of the Horizon in Photographed Outdoor Scenes by Human and Machine

**DOI:** 10.1371/journal.pone.0081462

**Published:** 2013-12-12

**Authors:** Christian Herdtweck, Christian Wallraven

**Affiliations:** 1 Department of Human Perception, Cognition and Action, Max Planck Institute for Biological Cybernetics,Tübingen, Germany; 2 Department of Brain and Cognitive Engineering, Korea University, Seoul, South Korea; University of Muenster, Germany

## Abstract

We present three experiments on horizon estimation. In Experiment 1 we verify the human ability to estimate the horizon in static images from only visual input. Estimates are given without time constraints with emphasis on precision. The resulting estimates are used as baseline to evaluate horizon estimates from early visual processes. Stimuli are presented for only 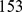 ms and then masked to purge visual short-term memory and enforcing estimates to rely on early processes, only. The high agreement between estimates and the lack of a training effect shows that enough information about viewpoint is extracted in the first few hundred milliseconds to make accurate horizon estimation possible. In Experiment 3 we investigate several strategies to estimate the horizon in the computer and compare human with machine “behavior” for different image manipulations and image scene types.

## Introduction

Imagine browsing through the hundreds of images, taken for example during your last vacation, in search of one where you lean against a tree or one with a view of the village from the neighoring hill. Humans have no trouble completing this task effortlessly in at most very few minutes. Computers, however, still have great trouble finding an image given only such a semantical description, which is no surprise given that they have to interpret nearly every image: find and identify objects (you, a tree, the village), estimate viewpoint (from the hill), etc. We extract all that information in only a few hundred milliseconds per image. The analysis is not very thorough but detailed enough to form a superficial representation of the image content in our mind. This representation is referred to as the *gist* of the scene ([1]–[3]). It is defined as the first impression of a scene, which forms before the first saccade, so from at most 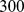 ms presentation time, and has been theorized to contain information about the scene type (beach/restaurant/forest/…), prominent objects in the scene (e.g., two people in the foreground but not the sailing boat in the distance), a rough geometric layout of the scene and the viewpoint.

Creating such a representation of an image might be beneficial not only as a basis for human visual processing but also for computer vision applications such as object detection, estimation of the 3D geometry of the scene, etc. It might help to solve problems often encountered in such applications like the lack of initial estimates for the depth of image structures or in the chicken-and-egg problems, that often occur in computer vision (e.g., segmentation and object detection).

There is a large body of work in both perception and computer vision literature dedicated to the aforementioned aspects of gist: human and machine object recognition and scene classification, (3D) space perception, geometry estimation. However, little is known about how humans estimate the last aspect, the *viewpoint*, although knowledge about this is often assumed in perceptual work and related cues are used in computer vision applications.

One aspect of viewpoint, that is mentioned most frequently, is the *horizon* of an image. Since this is also a quite intuitive measure of viewpoint we use this measure in our work as well.

We present three experiments on horizon estimation, two psychophysical and one computational.

In the first experiment we obtain ground truth data on the “estimatability” of the horizon in our stimulus set. Participants are asked to estimate the horizon heights in images, given enough time for careful decisions. The resulting distributions of horizon heights are then used as ground truth measure for experiments 2 and 3.

In Experiment 2 we validated theoretical considerations in the literature ([4]) that the gist of an image might contain information about the viewpoint. Such considerations seem reasonable since object presence, geometric arrangement and even scene type of an imaged scene are closely related to the viewpoint of the camera. Since the gist is extracted by early visual processes before the first saccade, a horizon estimate or related viewpoint information must be available after 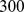 ms. We therefore had participants perform the same task as in experiment 1, but this time with only one very short (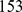 ms) presentation of each stimulus. We chose this time to make a thorough image analysis impossible and to limit participants' ability to memorize the image, so they had to rely on their first impression of the image for horizon estimation. We also measured the influence of several image manipulations on estimation performance.

In our last experiment we investigated the role of different cues to horizon estimation. We create several simple computer vision algorithms that estimate the horizon using different single cues. This could not only help in mimicking horizon estimation (and possibly gist) in an artificial vision system. Comparing the behavior of our algorithms with human results for the same image manipulations and same data set, might also give hints to what cues the human visual system might be using.

Some results of experiments 2 and 3 have been published in a conference article ([5]). However, we only compared estimates to a “ground truth” horizon estimated by the authors and limited the analysis of computer vision algorithms to the luminance channel. Furthermore, we could not present results from all the evaluations we performed due to page number limits.

### Viewpoint and Horizon

**Figure 1:**
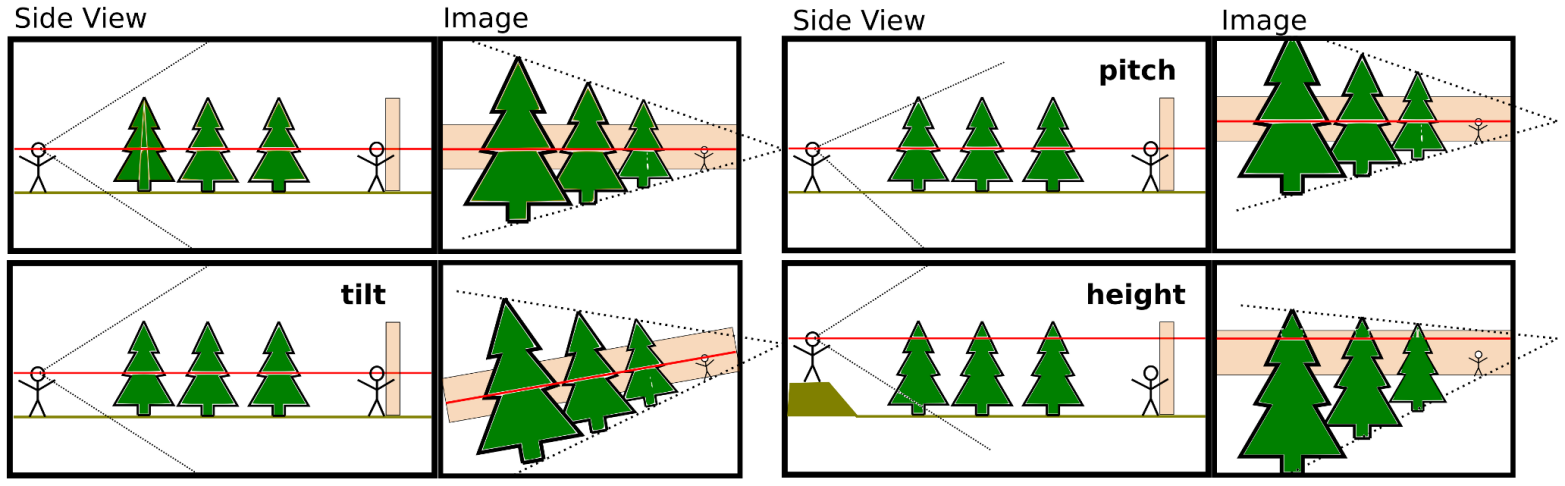
Visualization of changes in viewpoint parameters and their effect on the horizon in the image. For each plot the left part shows a side view of a simple scene while the right part shows the scene through the left person's eyes. The red line in the side view indicates eye height, the red line in the image is the horizon. The black lines in the left part indicate the viewing frustrum of the viewer, the black dotted lines on the right indicate parallel lines in the real world that converge on the horizon **Top left**: Reference scene with viewer looking straight ahead with head upright and from ‘normal’ eye height. **Top right**: A change in head pitch of the left person results in a vertical movement of all the image including the horizon. **Bottom left**: Roll, i.e., tilting the viewer's head to the side will rotate the whole image including the horizon **Bottom right**: If the left person views the scene from a higher position, the position of objects with respect to the horizon changes, the further away the object the maller the change.

**Figure 2:**
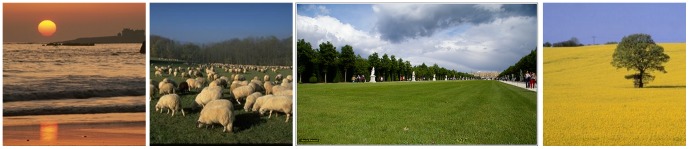
Images to explain horizon cues. **Left**: In the left half of the image one can see where sky and ocean meet, so the horizon must be at that height. **Middle left**: assuming the the ground has no slant towards the forest in the back, the horizon can be estimated by imagining a person right in front of the trees and pointing at that person's eye height. **Middle right**: a case where perspective gives a clear horizon estimate (parallel lines are the borders between grass and path or the tree tops). Note that this is not at other people's eye height, presumably the picture was taken from a sitting/kneeling position. **Right**: an example where the surface slant is hard to estimate, so the horizon could be anywhere below the sky. The tree might be seen as standing slightly higher than the viewer, so can give a lower upper bound.

Since “horizon” is a term widely used in different meanings, but often only explained by “where the sky meets the ground”, we explain the term in more detail here. Participants in our experiments received similar explanations, together with visualizations that are shown in figure 1 and examples in figure 2.

The astronomical horizon (in the rest of this work simply referred to as the horizon) is defined by the “horizon plane”, a plane that is perpendicular to gravity and located at the same height as the viewer's eyes/camera. In any image taken by this camera/viewed through these eyes, the plane is only visible as a horizontal line, which is the horizon line we are referring to in the following. It is not dependent on the slant of the ground surface nor on the presence of occluders (c.f. figure 1) — it is always orthogonal to gravity.

The *visible* horizon is the (usually not straight) boundary line above which only sky can be seen and below of which there is no sky (except, of course, reflections or smaller patches). This is usually above the (astronomical) horizon, except if the observer is standing on a highly elevated place. The horizon lightly differs from the *true* horizon, which takes into account the fact that the earth is not flat.

Further horizons have been defined in the psychophysics literature. The *terrestrial* (sometimes also referred to as *truncated*) horizon for example refers to the image height of the point on the surface plane that is furthest away from the viewer (which for finite surfaces without slant is slightly below the horizon). On can also extend the definition of horizon to the *horizon of an arbitrary plane* which is the line in the image where all parallel lines within this plane meet. This kind of horizon only depends on the plane's slant and coincides with “our” horizon for horizontal planes. There is also the *morphological* horizon which is the straight-ahead direction with respect to one's own body, so it is independent of the visual stimulus and only depends on the viewer's body orientation.

The most general rule to find the astronomical horizon in an image is, that everything that is above the viewer's eye/camera (and consequently above the horizon plane) is above the horizon line in the image, and the same holds true for things below the viewer/camera. The description of horizon as “where sky and ground meet” is true if the ground is sufficiently flat and there are no occluders, as is the case for many coastal scenes (e.g., figure 2, left). However, usually there will be buildings, plants or mountains occluding that line, or the ground surface is slanted, so it must be estimated. One cue in that case is to estimate where on the occluder the viewer's eye height would be. In case of other people of similar height on a flat ground this is easy, the horizon must be close to their eyes as well. For other occluders one must estimate their distance and size to find the viewer's eye height there (see figure 2, middle left). If the occluder is very far away, then its ground contact point (i.e., the terrestrial horizon) is a good approximation to the horizon because the own eye height is negligible in the distance (imagine the height of people on the island in the left image of figure 2). Another clue to the horizon height is *perspective*. Horizontal lines that are parallel in the real world will meet in the vanishing point which is on the horizon. This can be used in structured environments like cities or parks where often many parallel lines are present (as is the case in the middle right image of figure 2). This has also been referred to in the literature as the *parallel lines* and the *horizon rules* ([6]). All these clues (except the “above stays above”-rule) fail, however, if the ground is not flat. In that case one can try to account for the effect by estimating the amount of ground slant or rely on other senses, gut feeling and heuristics (see for example figure 2, right).

The horizon is influenced by most other aspects of the viewpoint as illustrated in figure 1 for pitch and roll of the camera as well as change of viewer height.

Although the proper definition of the horizon sounds very theoretic, it is a measure that is quite intuitive once participants were familiarized with the practical application of horizon estimation as described above.

In real-life situations, additional sources of information can be used to estimate the horizon: these include dynamic visual cues such as optical flow, proprioceptive, and vestibular cues. Final horizon estimates are likely formed by combining estimates from all these input sources. Here, we concentrate on horizon estimation from static pictures of outdoor scenes in the context of the aforementioned prior work on scene gist ([1]–[3]).

### Related Work

The horizon is a factor that is used quite extensively in the literature on space perception and self-orientation as well as computer vision. However, very little work has been done on how humans actually estimate it. We will therefore summarize the literature in the mentioned fields with a bias towards horizon estimation. We also point to literature on early visual perception and computational methods used for viewpoint estimation.

#### Self-Orientation

In order to identify the horizon in the world one needs to determine one's body, head, and eye orientation with respect to gravity. A long line of research has been devoted to the estimation of self-orientation, early examples being the observation by Aubert [7] that in a dark room the apparent orientation of a line changes with body sideways tilt, and the famous experiment showing that a tilted room affects the perceived upright direction [8], [9]. Gibson, who later emphasized the importance of the horizon for visual perception [10], also contributed in his earlier work to this line of research by describing the effect of gravity and strong motion on the otherwise very stable perception of the horizontal and vertical [11]. Newer studies on self-orientation perception, relevant for horizon estimation, are concerned with estimation of body pitch. Cohen and Larson [12] strapped participants to a bed that could be pitched by a motor and asked them to adjust their pitch to certain orientations which produced angular errors up to 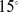. In the same study participants had to estimate the horizontal component of the straight-ahead direction relative to their body (the morphological horizon) which they could do with a precision of around 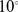. Matin and colleagues [13] determine the importance of extra-retinal eye information for estimation of the horizontal in darkness by paralyzing the eyes with curare. The higher precision of an estimate of the horizontal given visual stimulation as compared to darkness was shown in many studies, for example in [14], where participants had to adjust a chair's height until a illuminated target appeared at eye level. A long line of research was performed by Matin and Li on estimating the visually perceived eye level (VPEL) i.e., the straight-ahead direction with respect to true gravity given a pitch of the observer, a room of various pitch angles and different lighting conditions (darkness, whole room, degraded line stimuli), which they summarized in the great circle model [15]. Other studies analyzed the effect of gravity [16], [17], gymnastic expertise [18], gender [19] or response strategies [20] on the estimation of the body-referenced or gravity-referenced straight-ahead.

#### Space Perception

The orientation of one's body affects and is affected by the percept of the visual surrounding, in particular the visual horizon. There is therefore quite some literature in space perception that assumes an estimate of the visual horizon (often in form of the horizon of a ground plane) or the horizontal direction and uses it to explain our perception of distance and height of objects and slant of surfaces. An early overview over this field was given by Sedgwick [21]. More recent work includes the study by Rogers [22] on the horizon-ratio relation [10], [23], which states that the horizon line in an image intersects objects standing on the ground at eye height, which can be used to estimate their height. From simple line displays participants estimated the height of objects with high precision if the horizon line was close to the middle of the display. Otherwise performance probably dropped because participants perceived the line not as the horizon but as an edge of an object. Even without any hint on a visual horizon participants made strong assumption on the perspective in the image which shows the presense of a strong bias on perspective in images. Ozkan and Braunstein [24] conducted a similar study and found high agreement between relative distance estimates of two ellipses in front of a simple line drawing with distance estimates of cylinders rendered on top of photographs with clearly visible horizons. They also examined the influence of the ground and ceiling surface and an explicit vs. an implicit horizon, finding that both the implied vanishing points of ground and ceiling surfaces as well as the height where the ground surface terminates influence horizon estimates. The effect of ceiling vs ground plane was also a topic of the study by Thompson and colleages [25] with the result that the accuracy of blind-walking to targets on the ceiling and ground plane were surprisingly similar but are affected by modifications of the horizon height.

The measure that is most directly linked to the horizon is angular declination or elevation of an object, which is the angular difference between the straight-ahead direction (i.e., to the horizon) and a line from the observer's eye to an object's ground contact point. In a study by Philbeck and Loomis [26] angular declination was the most informative cue for distance estimation. Ooi, Wu and He found in severla studies that angular declination is estimated quite accurately [27], that a change of angular declination changes estimated distances [28], that these estimates depend on a scanning of the ground surface [29], and that for judging the straight-ahead direction the ground surface parallel lines are used [6]. The latter study also showed that for judging the straight-ahead direction information from the ground plane is more important than that from the ceiling [6]. This might be due to a higher importance of regions below the horizon for estimating the horizon position, which will also be tested in experiment 2. Studies in VR [30] and with degraded viewing conditions [31] further underline the importance of horizon for distance estimation.

There are many more cues in literature for estimating the horizon including motion information [8], [21]. The strong regularity of the horizon has also influenced animal and human physiology [32], [33] and biases our saccade direction and more generally the way we take and look at photographs [4].

### Computer Vision and Graphics

In computer vision the horizon is often used as a hidden variable that is determined through indirect measures and affects the image interpretation. In the work by Hoiem et al. [34], [35] the horizon height in the image is estimated using converging lines, Gaussian priors, and known real-world heights of objects to estimate the height of detected objects. Converging lines have also been used by Kosecka nad Zhang [36] to estimate the camera orientation with respect to a roughly planar world. In [37] as well as [38] the authors follow a similar goal, replacing traditional edge detection with probabilistic models that elegantly pool edge evidence over the image.

Finally, Torralba and Sinha [39] have proposed to use the GIST descriptor [1] of an image as feature for horizon estimation. They trained a mixture of linear regressors on the GIST feature to guide object detection. This is also used in [40], [41] to create image compositions with correct perspective. We use this descriptor in our computational experiment.

## Methods

### Ethics Statement

The experiments described later in this manuscript use human volunteers. Informed written consent was obtained prior to any experiment or recording from all participants. Participants and data from participants were treated according to the Declaration of Helsinki. The experiments were approved by the local ethics committee of the University of Tübingen (Project number: 89/2009BO2).

### Ground Truth

In order to evaluate the performance of human participants and algorithms we need stimuli for which the true horizon position in the image is known. There are several ways to obtain such data. Some new camera models have integrated sensors that measure the camera pitch. Unfortunately that information is only visualized in the camera display, but usually not saved in electronic ways. Another possibility is to used rendered images of artificially created 3D scenes as stimulus set. This would allow for very controlled manipulations of the images at the expense of naturalness of the stimuli. Both these methods would provide us with the true, physical position of the horizon in the scene. We are, however, interested in a purely *visual* estimate of the horizon which might be very uncertain or even different from the physically true position. Given for example a surface with a slight slant and few other cues to hint at the slant, the true horizon is of course still at a well-defined single height in the image which can easily be estimate if one were in the scene. The horizon in the image, however, is not a single well-defined point because it depends on the viewer's estimate of the surface slant. In this case we will want to penalize a slight deviation in the horizon position less than in images with many cues to the true horizon position. We therefore decided to use existing, natural images and gather estimates from several participants to approximate a distribution of visual horizon “estimatability” over the image height in experiment 1, and use this as a reference for the evaluation of participant and algorithm performance in experiments 2 and 3.

### Stimuli

We restrict our stimulus set to landscape images of outdoor scenes, since the ecological value for horizon estimates in closer-up shots like portraits and indoor scenes is doubtful, and since horizon estimation in these cases might be hard or impossible. We selected images from two sources: the very large LabelMe image data base ([42], http://labelme.csail.mit.edu) and — since LabelMe has a bias towards man-made environments — a database of 3645 images from German zoos and wildlife parks (http://images.kyb.tuebingen.mpg.de). From LabelMe we considered all image folders that predominantly contained images from outdoor scenes (171 in 2008). To avoid further biases in the image selection process we chose random images from these two sets with the following restrictions: Firstly, they had to be free of noticeable camera roll, which is rarely encountered in everyday situations since humans usually avoid sideways tilt of the head. Secondly, images had to provide enough visual queues for horizon estimation, which given the multitude of cues to horizon estimation (see above) also poses no serious restriction on the stimulus material. In this subset we labelled and verified the horizon position in random order until we had 300 images with a roughly homogeneous distribution of our (twice verified) horizon labels in the middle third of the image. We will refer to these labels as ‘expert’ labels later on, they were only used for stimulus selection. The reason for choosing a distribution in the middle third is two-fold. First we wanted to avoid biases participants might have caused by avoiding to click close to the upper or lower image border. The second reason is that some of the image manipulations we tested would have sometimes rendered our expert horizon outside the displayed part of the image. Finally, we restricted the stimulus set to 300 images as more images would have resulted in excessively long experiment durations.

**Figure 3:**
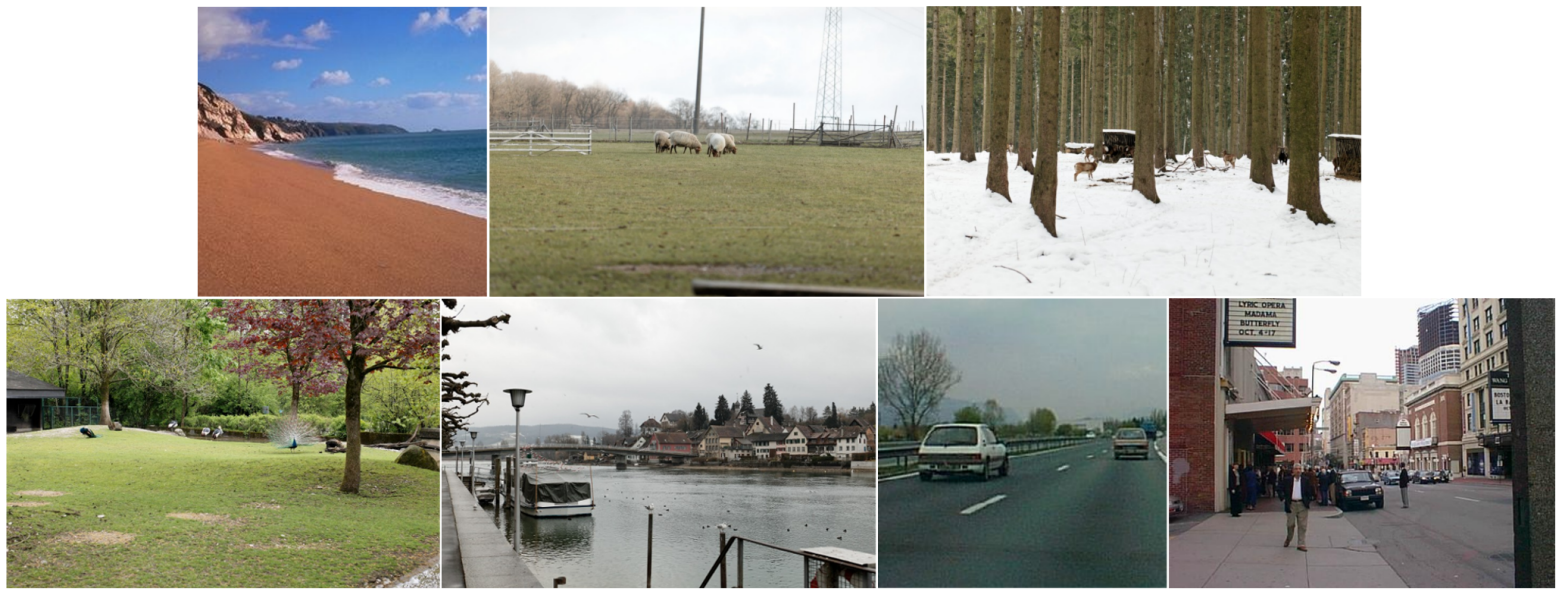
Example stimuli from every scene class. (roughly from natural to man-made): coast, open country, forest, enclosed natural scene, other, non-urban street, city.

To also study the influence of scene type on horizon estimation, we assigned each of these 300 images to one of seven scene types which are (roughly from natural to man-made): coast (32 scenes, mostly beach), open country (29 wide views of natural landscapes), forest (18 scenes dominated by trees which are quite close), enclosed natural scenes (46 images of open natural regions which are enclosed by forest and/or walls), non-urban street (16 views of streets in mainly natural landscapes), city (47 pictures taken in urban regions), and other (12 images that could not be assigned to any other class). Examples for each of these scene types are shown in figure 3. For experiment 2 we removed 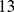 images from the stimulus set which were very similar to other images to reduce the number of trials.

To investigate the importance of different cues for human horizon estimation, we manipulated the images corresponding to the following six conditions:

**norm:** the original image as baseline

**inverted:** the image flipped (mirrored at the horizontal axis); this affects the holistic processing of the image leaving the structure itself and local features intact

the image is convolved with a Gaussian filter which removes low-frequency information leaving the holistic impression unaffected. The standard deviation of the filter was chosen manually as 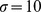 pixels for images with resolution 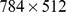 pixels, such that the rough image structure was still visible but fine details were more difficult to discern For images with different resolutions, the filter was scaled appropriately (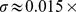 image size).

**lower:** the lower two thirds of the image are cropped, to study the importance of information from the lower image region for horizon estimation. To avoid participants noticing this manipulation through the change in image size or aspect ratio we removed image columns randomly from the left and right image border until the aspect ratio was the same as the original. The remaining image was shown with a bigger magnification to preserve visual angle

**middle:** we changed the image as in the lower condition except that we took one sixth of the image away from the top and bottom of the image, leaving the middle two thirds of the image

**upper:** the same procedure as for the lower condition, removing the lower third of the image to study the importance of the remaining upper two thirds of the image.

**Figure 4:**
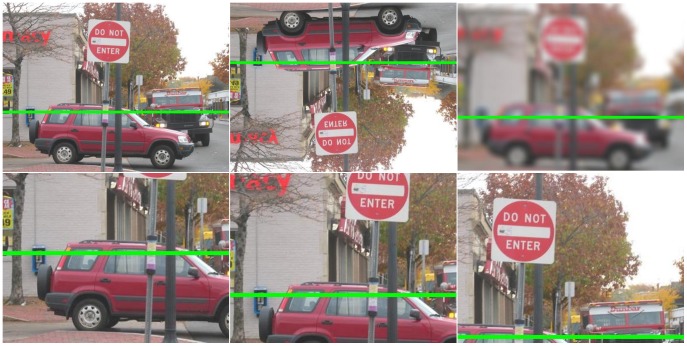
Examples of stimuli in the different conditions. : normal, inverted, blurred, lower subwindows, middle subwindow, upper subwindow; green bar is the expert estimate.

Examples for the six conditions for a single image are shown in figure 4.

Examples for the six conditions for a single image are shown in figure 4. One reason for including the three “subwindow conditions” (lower, middle, upper) was to diminish the photographer's bias that might favor certain objects and viewpoint compositions and that might give additional cues to horizon estimation. The only image region that is present in both, upper and lower subwindow conditions, is the middle third of the image. This is the second reason mentioned above why we originally chose stimuli with our expert horizon estimate in the middle third. In experiment 1 participants were only presented with the original images.

### Procedure for Psychophysical Experiments

Twelve paid participants (6 male, 6 female, mean age 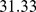 years, age std 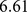 years) took part in experiment 1, twenty particpants (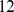 female, 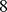 male, mean age 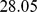 years, std 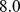) took part in the second experiment. They were informed that they would view the appropriate number of images and that they would be asked to estimate the horizon in those.

For experiment 1, participants were placed in front of a laptop with a standard LCD screen (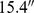-inch widescreen with LED backlighting) at an otherwise empty desk in a regular, empty office. Experiment 2 called for a more controlled setup since the very short presentation time required a high level of concentration. To avoid external distractions or influences of low-level factors like viewing angle on the results we placed participants in a dark room at a fixed distance of 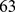 cm from a flat CRT screen (screen width 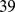 cm, viewing angle 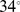, resolution 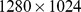 pixels, 85 Hz refresh rate), with their heads resting on a chin rest fixed in front of the screen roughly at the middle of the image.

Each participant of each experiment was carefully instructed about the horizon definition and cues to its estimation, using verbal explanations very similar to the one given in the Introduction above, using images shown in figures 1 and 2. Following the theoretical explanations participants were familiarized with the estimation user interface and performed test trials with increasingly difficult images that did not appear in the experiment. The experimenter discussed the first estimates with participants until they felt comfortable with task and interface. For later training trials, feedback was reduced to simple comments on correctness of estimated position and estimation speed. The experiment was started when participants had understood the task and estimated the horizon with reasonable precision in 10 images without instructor interference. The user interface used in the experiments was written in Matlab (The Mathworks, Inc., Natick, USA) using PsychToolbox 3.0 [43]. Images were presented in the middle of the screen, filling half its height with a 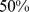-gray background. Each participant was shown all stimuli in a randomized order that was different for each participant.

**Figure 5:**
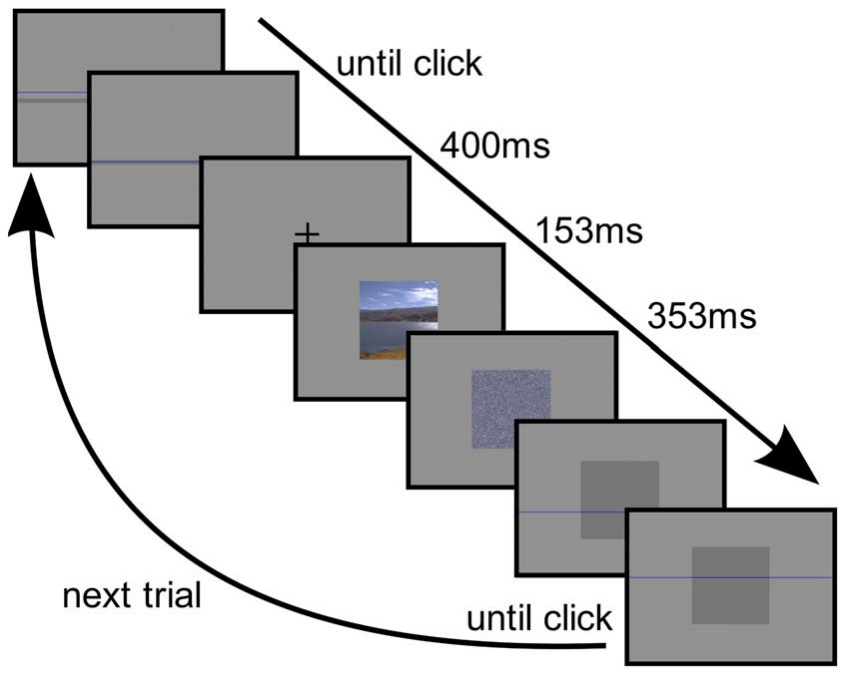
Chain of events in a typical trial. A dark gray bar appears, the participant moves the blue line into it and clicks; then a fixation cross appears for 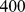 ms, followed by the stimulus which stays on for 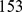 ms, and is immediately masked for 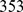 ms. Finally, the mask is replaced by a dark gray rectangle and the blue line re-appears which the participant moves to the position of the estimated horizon and clicks.

Each trial was started with a 50%-gray screen on which a “cursor” in form of a horizontal blue line spanning the whole screen width was visible. As a “start button” a dark gray bar spanning the screen width was shown at a pseudo-random height in the middle third of vertical region of the screen that contained the images. Participants started the trial by moving the blue line, which was controlled by the mouse, into the dark region and clicking left. The blue cursor then disappeared and instead a fixation cross was shown for 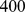 ms, followed by the stimulus. In experiment 1 this was displayed until participants had estimated the horizon position in (or outside of) the visible image region by placing the blue line and clicking the left or right mouse button. In experiment 2 the image was only shown for 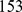 ms and then immediately masked by a pixel-scrambled version of the image for 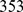 ms. Only then the blue cursor line re-appeared allowing participants to estimate the horizon on a dark gray rectangle of the same size and position as the stimulus. After participants clicked the left or right mouse button the dark gray “start button” bar for the next trial appeared. The vertical position of this bar was chosen to be at a minimum distance of 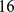 pixels from the estimated position to avoid the feeling of feed-back. We also emphasized that fact in the briefing because in a preliminary experiment some participants had interpreted the bar as feed-back in some cases. The procedure for experiment 2 is visualized in figure 5.

The short presentation time in experiment 2 made most cognitive strategies to horizon estimation impossible, forcing participants to rely on their first impression or “gut feeling”. To further enforce this participants were instructed not to try to be too precise but rather emphasize on speed. In contrast, participants in experiment 1 were instructed to respond as accurately as possible, emphasizing precision rather than speed to motivate a more careful analysis of the stimuli. For both experiments we told participants that if they could not estimate the horizon in the image they should place the horizon line at an arbitrary position and click the right instead of the left mouse button, indicating that they were not able to estimate the horizon in this image. Such answers could be due to a general inability to estimate the horizon in images, to distractions, or to lack of concentration during stimulus presentation. Positions from these “do-not-know”-replies were not included in the evaluation since they contain no relevant position information. The reaction time from such trials, however, was included in the evaluation, since a right-click is a valid response and the time to decide on it can be compared to times required to decide on a horizon position estimate.

We recorded the time from image presentation start until click in seconds, the position where participants clicked and whether participants clicked with the left or right mouse button. Estimated positions were normalized for height of the full upright image, so 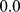 indicates the uppermost image row in the unmodified image, 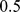 the middle of the image and 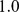 the bottom of the original image, already accounting for the condition to make estimates comparable between conditions. Results from trials where participants responded with a right click were discarded from the analysis of positions, but not from the analysis of reaction times.

One other major difference between experiments 1 and 2 was the number of trials. For experiment 1 each participant estimated the horizon in each image of the original 300 images. For experiment 2 each participant estimated the horizon of every image in the slightly reduced stimulus set, for every condition, resulting in 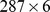 trials. To avoid fatigue we divided these into four blocks of 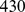 trials each and encouraged participants to pause for at least five minutes between blocks. Care was taken that there were at least 4 other images presented between the presentation of the same image in different conditions. Participants were also told that they could make minor breaks between (not within!) trials if they noticed a decrease in concentration.

As a preprocessing step for experiment 1 we removed outlier positions (not reaction times) from the analysis to avoid noise from manual errors in our ground truth. For this we calculated for each image the standard deviation across all participant estimates and removed all estimates that were more than three standard deviations away from the participant mean of the corresponding image. There were 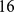 such cases (note that 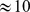 cases are to be expected given that for a normal distribution 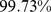 of the probability mass are concentrated between 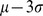 and 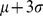).

For experiment 2, we did not exclude outliers. However, early analyses showed that two participants had not understood our instructions about the inverted condition. Their results were inconsistent with estimates in the inverted image. Instead their results for the inverted condition showed a remarkably good performance if they were applied to the upright images, suggesting that those two participants had performed a mental rotation or flipping of the image and clicked at the position where in the then upright image the horizon would be. Since we cannot predict what other influences this process might have had, we excluded all results for all conditions from these two participants from the analysis.

Participants of both experiments filled in a questionnaire after the experiment.

### Computational Experiments

We designed several algorithms that exploit different cues to horizon estimation in images, including those mentioned in the perception literature (e.g., [21]). Each of these algorithms gets a single image channel (e.g., the luminance) as input and returns for every image row a confidence value for that row containing the horizon. We chose a simple winner-takes-all framework to deduce from these confidences the algorithm's estimate. The only exception from this is the algorithm **[gst]** which does not give a confidence for each image line but directly returns the estimated horizon position. Each algorithm's parameters were optimized using excessive random search.

The algorithms are the following:

**[div]** is a very simple algorithm that uses a mixture of global and local features. It tests for each line how well it divides the image into a light region (i.e., containing high values) above and a dark region (i.e., containing lower values) below it by calculating the concentration of high values above and of low values below the given line. This is inspired by the fact that at day time the sky is lighter than the ground, so if applied to the luminance channel of an image this should yield the best separation between sky and ground. To allow for a more precise localization a weighted average of the (global) lightness concentration difference with the local vertical gradient at that line is calculated (after light smoothing of the image). A linear weighting of global and local features then yields an estimate for each line. Interestingly, parameter optimization resulted in a weighting that strongly biased the algorithm towards the local information, in essence making this algorithm quite similar to **[lin]**.

**[-div]** is the same algorithm as **[div]** except that light and dark are exchanged. The assumption that the region above the horizon is lighter than below might not be true for images taken at night time and especially might only hold for the luminance of an image, not for color channels.

**[lin]** works only on a local scale. It looks for horizontal lines in the image by calculating the vertical gradient in the image and summing its absolute value over image lines, assuming that around the horizon the differences between neighboring lines is greatest.

**[gab]** hinges on the same idea as **[lin]** : that around the horizon there should be the most notable vertical gradient. This algorithm, however, takes a more biologically motivated approach to finding these gradients, namely by convolving the image with a horizontal gabor filter which has been proposed as a good model for line-sensitive cells in L1 of the visual cortex. The gabor filter used is aligned horizontally with optimized wavelength, bandwidth and selectivity. After filtering in Matlab using code by Peter Kovesi (function spatialgabor from http://www.csse.uwa.edu.au/ pk), it sums for every line the magnitude of the response per pixel.

**[van]** uses the perspective cue most frequently proposed in the perception literature. It tries to estimate the vanishing point by finding image positions where many lines converge. In order to do this the Canny edge detection filter is applied to the image. A Hough transform is then applied to the resulting Canny edge image to identify lines and their strength, with the latter depending on the edge filter response and the length of the corresponding edges. The lines identified by the Hough transform are then added to a black (empty) image with an intensity value proportional to their line strength. Finally, lines are elongated to span the whole image. The resulting image 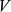 has bright spots in positions 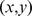 where many lines meet, corresponding to candidates for the vanishing point. Before summing up the strength of vanishing points 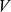 in each line 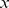, maxima are emphasized by exponentiation, resulting in confidences 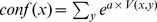. Parameters to optimize include the size of the edge filter and two thresholds for edges for the Canny algorithm, as well as the resolution of the Hough transform and the exponent 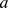 for exponentiation.

**[gst]** is the only algorithm we could find in the computer vision literature, that was explicitly designed to estimate the horizon of an image. It has been proposed by Torralba and colleagues in their work on scene understanding ([39]). It uses the *spatial envelope* as feature, the response of a set of filters tuned to different orientations and scales, which has been proposed as a psychophysically-motivated feature for scene classification. The algorithm does not calculate a confidence for each image line but instead returns a single estimate from a mixture of linear regressors which are trained on example images and corresponding horizon labels using an expectation-maximization procedure. We re-trained these regressors using our stimulus set and mean estimates in a cross-validation procedure. We also extended the algorithm to use all color channels and not just luminance. Therefore this algorithm is the only one applied to not just one color channel like the other algorithms but uses information from all channels simultaneously.

**[dum1]** and **[dum2]** are algorithms included to give a lower bound on algorithm performance, similar to the chance level in many experiment analyses. They “estimate” the horizon by random guessing, ignoring the image information completely. **[dum1]** draws estimates from a uniform distribution over an interval 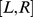 with 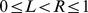 while **[dum2]** estimates from a normal distribution with mean and standard deviation 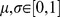. Parameters 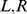 or 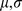, respectively, are identified during optimization.

**[exp2]** is included in evaluation plots to compare computational results on test images with those obtained from human subjects. Note that results may vary from those reported of experiment 2 because of the choice of images in the test set (see below).

Algorithms **[gab]**, **[van]** and **[gst]** were implemented in Matlab, all others in python. Evaluation and experiments were done using ipython (version 0.9.1, [44], python 2.6.2), using the packages numpy and scipy (version 1.5.1/0.9.1, http://scipy.org), as well as mlabwrap (version 1.1, http://mlabwrap.sourceforge.net), matplotlib ([45]) and OpenCV (version 2.1, http://opencv.willowgarage.com/). All images in all conditions were transformed into the CIE L*a*b* color space ([46]) and algorithms were applied to each of the resulting channels independently.

All algorithms have parameters that need to be optimized. To make the best use of our limited set of results for 300 images, we adopted a 10-fold cross validation procedure for training and testing our algorithms. Images are divided into 10 sets of 30 images by means of an optimization algorithm which tried to keep roughly equal ratios of scene classes and mean ground truth densities in all subsets. For each of the 8 algorithms, 3 color channels and each of the 10 image subsets 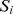 parameters are searched that best reproduce the human results on the 9 image sets 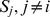 (their “training sets”) in normal condition. This results in 10 optimal parameter sets for each algorithm and color channel. As criterion for reproduction of human results we chose the value of the ground truth density derived from experiment 1 at the image line with the highest horizon confidence. The parameter search was done using first an extensive random search in parameter space followed by a refinement around selected maxima using the Nelder-Mead simplex algorithm (scipy.optimize.fmin).

As exceptions to this procedure, algorithm **[gst]** was not training on individual color channels but on the full L*a*b* images. Since evaluation of **[gst]** 's performance on a given image set for certain parameter involves training a regressor, a 3-fold cross validation for each parameter evaluation of **[gst]** had to be performed during the parameter search.

The performance of the final regressor — trained on all 9 training subsets with optimal parameters found on them — was averaged over all 10 folds and is reported in the following as training performance. Algorithms **[dum1]** and **[dum2]** were not trained on individual color channels since they do not “look” at the image at all. Instead confidences were averaged over 10 runs of **[dum1]** and **[dum2]** to account for the randomness.

To finally evaluate the algorithms' performance, we report in the Results section the performance of algorithms with optimal parameters on the left-out subsets which together make up all the original 300 images. For example, results on 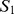 are computed with parameters searched using 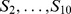. We also ran algorithms with optimal parameters on their respective training sets in other conditions.

## Results

### Ground Truth Experiment

**Figure 6:**
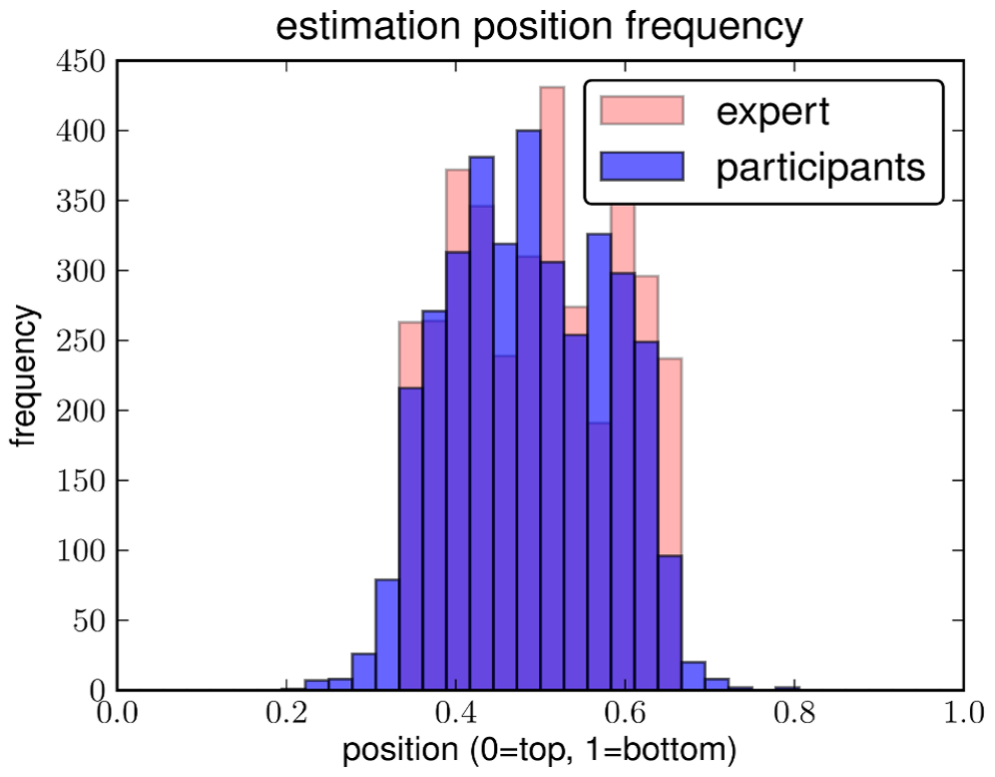
Agreement of results from experiment 1 with expert labels. **Left**: Histogram of relative image positions participants clicked at compared to the distribution of expert estimates by the authors. **Right**: correlation of individual subjects' estimates (black) and their mean (red) with the expert estimate. Both individual estimates and estimate mean show a very high correlations with the expert labels.

The distribution estimates from experiment 1 agreed well (correlation 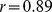, c.f. figure 6 left) with our expert estimates that were used to select the stimulus set. Estimates show a roughly homogeneous distribution of horizons in the middle third as can be seen from the histogram in figure 6 (right).

Participants were told to click with the right instead of the left mouse button in case they could not estimate the horizon in the image. This happened only twice in all of the first experiment, which can be explained by manual errors.

The agreement with our expert estimates and the low number of right-click results leads us to believe that the task was sufficiently clear and intuitive.

**Figure 7:**
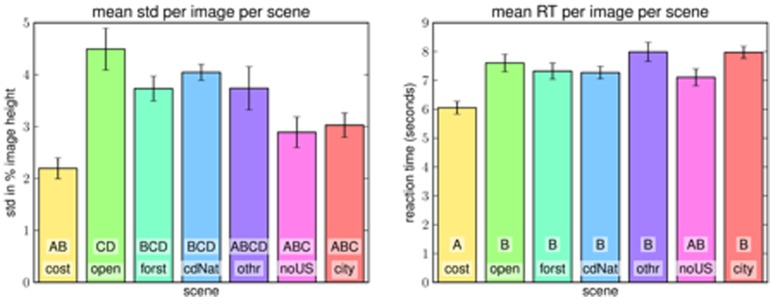
Reaction time and consistency of experiment 1 results with respect to scene type. **Left**: Standard deviation of participant results per image in the ground truth experiment, shown as average per scene type. Results of pairwise comparisons with the Scheffé criterion after a 1-way analysis of variance are indicated by letters A–D: first row denotes group, second bar shows letter of other groups, to which this group is not significantly different; “other” is not significantly different from any group. **Right**: mean reaction time of participants per image in the ground truth experiment, shown as average per scene type. Error bars denote standard error. Bars with letters A–B are significantly different from another. The class “non-urban streets” (noUS) is not significantly different from any other class.

Estimates agreed not only with our expert estimates but also with another. The standard deviation between participants for each image was 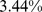 image height on average (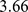 before removing outliers as described in the Methods section). This deviation between participants varied greatly with scene type. Figure 7 (left) shows that agreement on open coastal images is generally much higher (std of 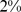 image height) than on more closed scene types like open country, forest or closed nature (all close to 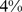 image height). Street scenes within cities (class city) and outside urban areas (class non-urban street) resulted in standard deviations of approximately 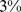 of the images' heights. We conclude that humans are able to estimate the horizon from a monocular image with reasonable accuracy, at least if given enough time.

The reaction time results show a much more homogeneous distribution with respect to scene type (figure 7 right). Participants in experiment 1 took 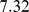 s on average to estimate the horizon. The most notable difference is again caused by the relatively easy images from the coast class (c.f. figure 2).

#### Density Estimates

**Figure 8:**
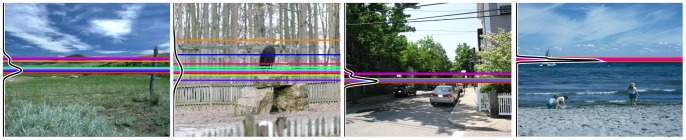
Examples of stimuli and results from experiment 1. Individual participant estimates are shown as colored lines, the black curves on the left of each image show the estimated estimate density function. Note how the variance of estimates varies across images.

The 12 estimates for each image (11 estimates for 2 cases of right-click images) are used in experiments 2 and 3 as ground truth distributions for the visual horizon. We chose this measure because it captures not only the position of the horizon but also the variability in estimates caused by the absence of non-visual cues. To measure how well other estimates agree with these estimates, we fitted a mixture of Gaussians to these “ground truth estimates” and report the value of the resulting probability density function at the position of the estimate to evaluate. The method for fitting a distribution to these estimates was scipy.stats.gaussian_kde. For a given image 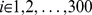 it approximates the unknown distribution from which samples (estimates) 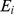 are drawn, by replacing each estimate 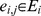 by a Gaussian distribution centered at 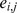 with a deviation given by the covariance of 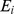. This yields a distribution with density function ([47])
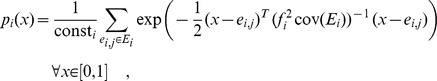


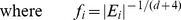
 is Scott's factor [48], 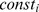 is a normalization constant, 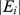 the number of estimates in 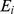 (typically 12), and 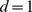 is the dimensionality of estimates. We call an evaluation of 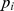 at a point 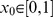 a *confidence* and the density 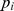 from which it was taken a *ground truth density* in the following. Since the ground truth densities have an integral of 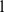 but are close to 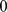 over most of the interval 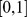 on which they are defined, point evaluations 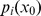 will often give values much larger than 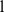. Examples of such density functions are plotted in black on the left side of example stimuli in figure 8.

### Psychophysical Experiment

Although 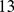 similar images were removed from the experiment and despite very quick reaction times, the 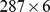 responses per participant took 2 hours 6 minutes on average (std 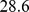 minutes) not counting time for breaks.

In this subsection results for experiment 2 are reported: Reaction times, confidence based on ground truth estimates from experiment 1, inter-rater correlation and mean position per condition, as well as a short analysis of training effects.

Our main measure of performance is the confidence as defined above: we evaluated the ground truth density obtained from experiment 1 estimates at the estimated horizon positions in experiment 2, resulting in a confidence which measures how well an estimate can be explained by the ground truth estimates from experiment 1.

The mean reaction time per trial was 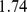 s (std 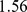 s). This is significantly faster than replies in experiment 1 (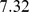 s), showing that participants followed a more intuitive and less cognitive strategy. This faster reaction time is not entirely explainable by time required for purely low-level visual processing, motion planning and execution. Although higher-level visual processing was severely limited by our masking paradigm, participants seem to still have processed the visual information to make their estimate.

Short-time memory might have played a role in two ways in this task. First, participants had to retain in memory either the horizon estimate they had already formed or some visual information of the image to base an estimate on. We cannot completely rule out that enough higher-level visual information had been retained to employ more cognitive strategies to the task, although we tried to minimize the probability of this by showing a mask after each stimulus in an attempt to purge the visual short-term memory of the image. For the effect of memory on a longer time scale, see the section on training effects below.

Correlating the distance the mouse was moved with reaction times showed that the time for mouse motion was not an important factor in reaction times.

#### Subjective Confidence

The first question we were interested in was, whether participants felt they could perform the experiment i.e., whether it is feasible to estimate the horizon from such a short presentation time. This seems to be the case as only 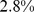 of all replies were a “do-not-know” indicated by using the right instead of the left mouse button to reply. Another indicator is the reply participants gave to our questions in the debriefing questionnaire. We asked them how certain they were about their estimates on a scale from 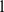 (very uncertain) to 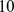 (very certain), which resulted in a mean response of 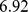 with a std of 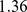. We also asked how difficult the experiment was (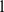 easy, 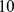 hard), for which we got 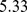 as mean reply (std 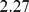).

#### Reaction Time per Condition

**Figure 9:**
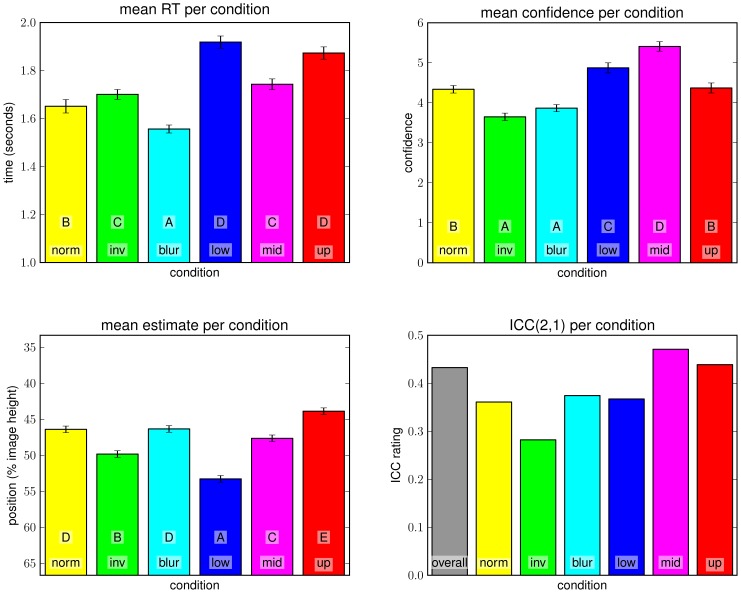
Results of experiment 2 per condition. **Top left**: Mean reaction time per condition. **Top right**: Confidence assigned to estimates per condition. **Bottom left**: Average position of estimates per condition, normalized to 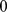 = image top, 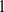 = image bottom. Although images were the same, positions vary significantly. **Bottom right**: Agreement of subject ratings as intraclass correlations ICC(2,1) per condition. In all plots, error bars denote standard errors. Bars with different letters A–E are significantly different according to a post-hoc comparison using the Scheffé criterion after 1-way analysis of variance.

As shown in figure 9 (top left), the reaction time per condition shows an interesting effect: We would have expected the fastest reaction time for the normal condition but participants replied significantly quicker for the blurred version of our images (Scheffé test with 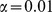). This may be explained by the lack of detail in those images that made attempts at careful and exact and therefore slower placement of the cursor impossible. Participants may therefore have felt less need to invest time for more precise cursor placement. This might have led to worse estimation results as shown in figure 9 (top right). Leaving out image information from top and/or bottom of the image, as well as image inversion, also slowed down participants significantly.

#### Confidence per Condition

Regarding confidence per condition (c.f. figure 9, top right) the worst performance for this measure is obtained from inverted and blurred images, which affect holistic processing in the first and high-frequency detail in the second case. Both these sources of information seem to be relevant to the task of visual horizon estimation. Again, the normal condition is not the one with the best performance. The lower and especially middle subwindow condition led to estimates that agree better with results from experiment 1. One possible explanation for this concerns all three subwindow conditions: participants saw less of the image, so there were less possible regions to falsely estimate the horizon at. The region around the horizon, however, filled a larger range of the displayed stimulus, thus offering more detail of the region around the horizon. A middle bias of participants would explain the performance increase in the middle condition but should also result in a smaller performance increase in lower and upper subwindow conditions, rendering these two the same level between the normal and the middle subwindow conditions. This is not the case, so this can not be the only explanation. We would rather suggest that information near the horizon, especially just below it, is most important for estimation. The fact, that the performance in the lower subwindow condition is much better than in the upper subwindow condition might hint at the ground dominance which has also been reported in the literature ([6]).

#### Position per Condition

Although every image was shown in every condition and although participant responses were normalized to positions in the full upright image, tests showed a significant difference between mean estimation position over different conditions (see figure 9, bottom left). Participants estimated the horizon in a lower position for the lower subwindows condition than in the middle subwindow condition, and even higher in the upper subwindow condition. There are at least two possible explanations for this behavior: Participants may have had a bias toward the middle of the visible part of the stimulus. In the lower subwindow condition, the middle of the visible part of the image is not at height 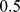 but at 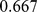 of the full image (0 = top, 1 = bottom). Also, the horizon estimation process could include a rivalry between a horizon estimated from the upper part of the image (a “sky-based” estimate) and another estimated based on the lower image part (a “ground-based” estimate), e.g., the lowest visible sky-pixel-position versus the highest visible ground-pixel-location. Removing information from the upper image part may result in a less confident estimate of the horizon from the upper image part and therefore to an estimate that tends more towards a “ground-based” estimate. The overall tendency in the experiment to a horizon estimate slightly above the middle of the image might be due to the stimulus set. However, there might also be a tendency of participants to click a bit too far up in the images, which could partly explain why the mean estimate in the inverted condition is lower. However, if this was the only influencing factor, the inverted condition would have to be exactly as much below 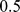, as normal and blur are above 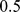.

#### ICC per Condition

Our final measure to evaluate agreement of participants uses the intraclass correlation coefficient ICC(2,x) ([49]), which measures the reliability of the ratings of several judges that all judge the same set of items. There are two version of this measure: the first, ICC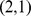 computes the reliabilities for each item separately, whereas ICC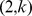 computes the reliability across averaged ratings. To compute these quantities we had to omit images in conditions for which at least one of the participants replied by a right click (“do not know”). If trials from all conditions are treated as independent items to rate, the overall reliability of participants is ICC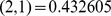 (based on 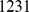 trials). The overall reliability of the resulting ratings is ICC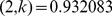. Both values indicate substantial agreement across raters ([49]). Reliability of raters split across conditions are shown in figure 9. Reliability for the inverted condition is much smaller than for the rest, while the middle subwindow condition show the highest reliability. Interestingly, here the upper subwindow conditions caused more consistent participant performance than the lower subwindow condition.

#### Results per Scene Type

**Figure 10:**
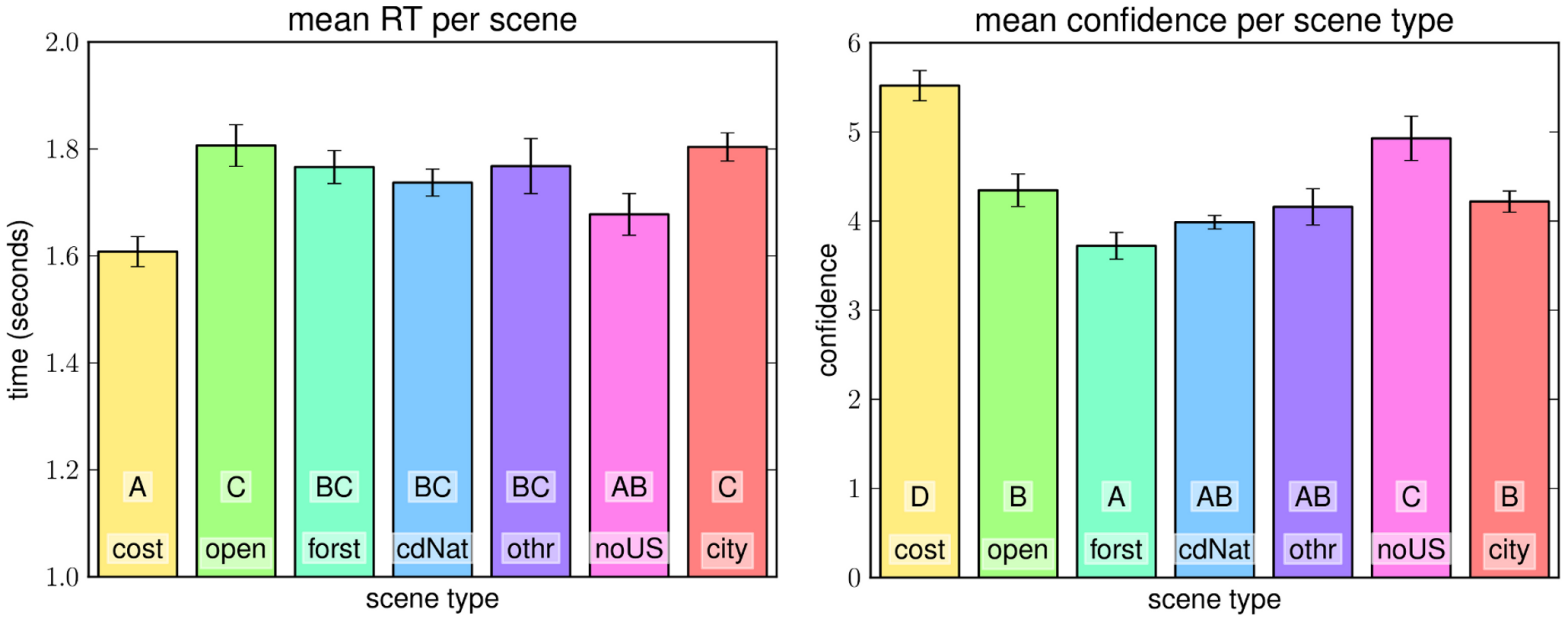
Results from experiment 2 per scene type. **Left**: Mean reaction time per scene type. **Right**: Mean confidence per scene type. In all plots error bars denote standard error; bars containing letters A–D are significantly different from all bars that contain none of the same.

As expected, participants responded quickest and most accurate for coastal images (c.f. figure 10). The next easiest class seems to be non-urban street scenes (labelled “noUS” in figure 10 Images of this scene type often offer wide views as well as perspective queues from the streets themselves or objects like cars or small houses. City and open country images result in the highest reaction times but not the worst confidences. This might be a hint at cognitive strategies being used more in these cases. Closed natural scene like forest and closed nature yield the worst confidences with respect to scene type despite relatively long processing.

Interestingly, the pattern of reaction time per scene in experiments 1 and 2 are very similar. Some degree of negative correlation between standard deviation of estimates from experiment 1 and confidence of estimates in experiment 2 is to be expected: if the standard deviation in experiment 1 is high, then the fitted distribution will attain smaller values distributed over a bigger region of the image. An estimate in experiment 2 that completely agrees with an estimate of experiment 1 will therefore be assigned a lower confidence than would be the case if the standard deviation in experiment 1 had been low. A relatively bad estimate, on the other hand, has a better chance to get some confidence in more diverse images, but in these cases the confidence is low enough to not make much difference in the mean. An interesting case in this respect is the open country class. According to experiment 1 it is the scene class for which fixing a ground truth horizon is hardest. Still, in terms of confidence in experiment 2 it outranks the classes forest, closed nature and other.

#### Training Effects

Since participants viewed each image six times, it may be possible that memory for the image could facilitate estimation in later trials. A few participants reported having realized that they had seen images repeatedly in different conditions. In those cases, recognition and retrieval of memory of earlier estimates could have had an influence on reaction time and estimated position. This should have no effect when comparing results within experiment 2 since the order in which images and conditions were presented was randomized. However, for comparing results of experiment 2 with those from experiment 1 and 3, a potential training effect should be taken into account. We therefore analyzed reaction times for experiment 2 estimates for the presence of training effects. In order to do so, confidences were re-ordered such that all trials, in which a subject saw an image for the first time (independent of condition), form one group, all second views a second group, etc. An analysis of variance of the resulting 6 groups means showed significant differences (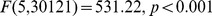), and a post-hoc comparison using the Scheffé criterion revealed speed-ups between all six groups from a mean of 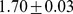 s (std err) for the first view to 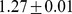 s (std err) in the sixth. The most likely explanation for this result is the increased proficiency with the task that caused participants to answer faster in later trials. The result, however, on its own cannot rule out a speed-up of estimation due to memory effects.

A similar analysis for estimated confidences, on the other hand, showed no significant differences in the group means 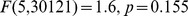. In particular, estimation performance was similar for the first time an image was presented (mean confidence 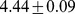 (std err)), and the sixth time (mean confidence 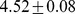 (std err)). Hence, although there were was a clear speed-up, trial repetition did not affect estimates themselves, which speaks in favor of a relatively stable rating performance.

Overall we conclude from the subjective participant confidence, the overall high agreement of estimates with the ground truth estimates from experiment 1, the high agreement between participants, and the lack of clear training effects in position estimates, that *horizon estimation is possible from simple visual processing* and that the horizon or related information could therefore well be part of the gist.

### Computational Experiments

We describe here some of the computational results obtained by applying the simple horizon estimation algorithms described above to luminance, a* or b* color channels of the stimulus set. We measure results first like in the psychophysical experiment by evaluating the ground truth density from experiment 1 at the estimated horizon position. This allows a comparison with human results (shown in black). To test whether algorithms “behave” like the human participants, we also calculated correlation coefficients of algorithm results with respect to condition and scene type as described below.

#### Confidence

**Figure 11:**
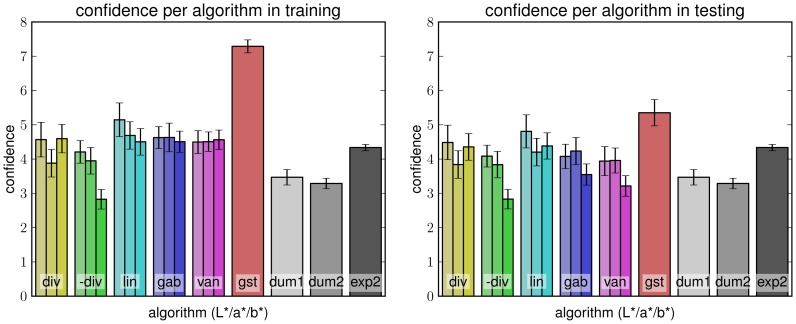
Algorithm confidence in training and testing. Mean confidence of algorithm results for normal condition with respect to densities from ground truth experiment in training (left) and testing (right); error bars denote standard error.

Our first measure for evaluating algorithm's performance is the same as used in experiment 2: confidence assigned by calculating the agreement of the estimate with those from experiment 1. We first tested whether algorithms were able to capture the information contained in the training set during parameter optimization. When evaluated on the *training* set (left plot in figure 11) we see that most algorithms managed to achieve the human performance from experiment 2. A multiple comparison analysis comparing all results with all others using the Scheffé criterion found no algorithm significantly different from **[exp2]**. Performing the same analysis using Tukey's honestly significant difference criterion, which is less conservative, identifies statistical differences from **[exp2]** for algorithm **[gst]**, and the two worst-performing **[dum2]** and **[-div]** (b* channel). The reason for **[gst]** standing out may be the relatively high number of parameters that allow a good fit to data. Since we used the training data itself, however, this analysis bears the risk of overfitting.

In *testing* in the normal condition (right plot of figure 11), the advantage of **[gst]** diminishes, still leaving it the best-performing algorithm considered here. However, its agreement with the ground truth densities from experiment 1 is nearly matched by **[lin]** and **[div]** for the luminance channel. This is confirmed by again comparing all results with all others using Tukey's honestly significant difference criterion. **[gst]** is significantly better than all algorithms except **[lin]** (L* and b* channel) and **[div]** for the L* channel. Luminance information seems to be the more informative for horizon estimation algorithms than color information. Interestingly, the worst performing algorithms here are using information from the b* channel, showing worse performance even than **[dum1]** and **[dum2]**. Significant differences from the worst algorithm **[-div]** (b* channel) are found for all algorithms except for **[gab]** and **[van]** (b* channel) and intelligent guessing ( **[dum1]** and **[dum2]** ). When compare to **[exp2]**, significant differences are found only for **[gst]**, **[dum2]**, and the b* channel-versions of **[van]** and **[-div]**. This is surprising since the b* channel is required to separate blue (like sky) from yellow (contained in sand and dirt), which would be expected to be of interest for horizon detection. Maybe the presence of green (i.e. low values for the a* channel) are more informative. Further analysis e.g., of statistical properties of image color distributions, might be of interest here.

#### Correlation of Behaviour

**Figure 12:**
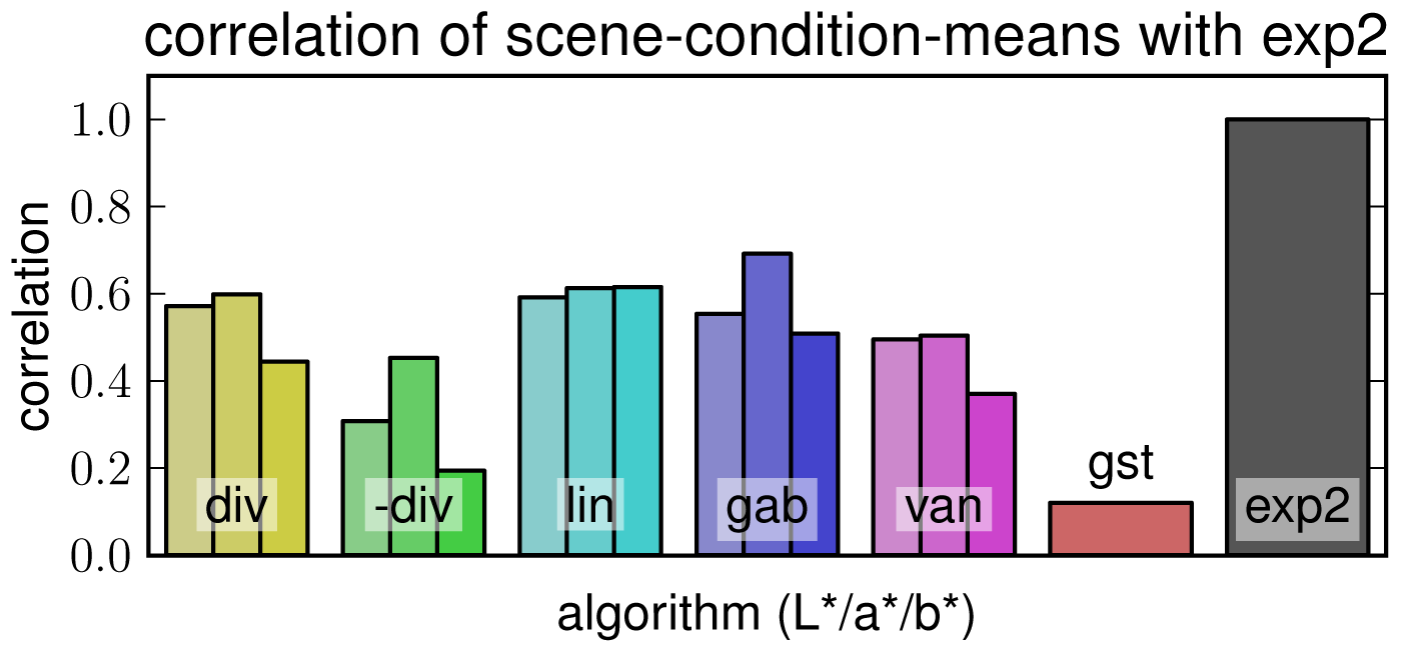
Correlation of algorithms' ‘behavior’ with human results. Bar height shows the correlation of pattern of confidence means per condition and scene type with human results from experiment 2.

We further correlated algorithms' ‘behavior’ i.e., pattern of mean confidences per condition and scene type, with human results. Since our ground truth experiment did not provide results per condition we correlated the mean confidence of algorithm with the mean confidence assigned to the human estimates in experiment 2 (i.e. with the corresponding results in **[exp2]** ). The results are shown in figure 12. **[gst]** shows remarkably bad correlation with results of participants in the quick experiment, although its performance when compared to experiment 1 ratings was highest. It seems to “behave” more like a carefully thinking human than a person that has seen the scene only very quickly. **[lin]** shows not only very good confidence but also the best correlation here, while **[-div]** produces low values in both tests. Over all algorithms, the a* channels slightly outperform L* and b* information, with a* of **[gab]** exhibiting the best correlation in this test.

## Discussion

Estimation of the viewer's orientation with respect to a viewed scene is an important task in human visual perception as well as computer vision. Although many studies in both fields assume some viewpoint knowledge in the processing system, it has not been explicitly tested how and how fast this knowledge is acquired. Literature on early vision suggests that simple viewpoint information like the horizon may be part of the gist, the representation of a visual stimulus that forms in the viewers mind within a few hundred milliseconds.

We have presented here three experiments addressing the ability to estimate the horizon in human and machine. We found that humans are well able to estimate the horizon from purely visual input in a consistent manner. Performance depends on the type of image displayed, with coastal scenes being the easiest to interpret. When forced to rely on only the first visual impression from a masked presentation of only 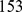 ms, performance drops slightly but still shows remarkable agreement with estimates from longer viewing times. We conclude therefore that viewpoint information is part of the first visual impression of an image.

By manipulating possible feature channels in the shown stimuli, and by comparing human estimation results with those created with simple, single-feature image processing algorithms, we addressed the question what information is used to estimate the horizon. Results from the psychophysical experiments suggest that high-frequency information plays no crucial role in estimation and that image information close to the horizon is more important than that in higher or lower image regions. Estimates based on information below the horizon show significantly higher agreement with estimates based on longer viewing times which is consistent with the prediction in the literature [29]. However, agreement between participants is higher for estimates based on the upper two thirds of the image, than those based on the lower two thirds. It seems, therefore, that both global and local processing are necessary for horizon estimation.

Computational studies show a similar trend: the **[gst]** algorithm, which estimates the horizon from a holistic representation of the image, exhibits the best agreement with human estimates from longer viewing times. However, its pattern of “behavior” with respect to changes in scene type and stimulus condition is least correlated with human results from short presentation times. Simple horizontal gradients perform relatively well in both evaluation criteria, consistent with simple local visual processes.
